# Laparoscopic Resection of an Intra-Abdominal Esophageal Duplication Cyst: A Case Report and Literature Review

**DOI:** 10.1155/2015/940768

**Published:** 2015-03-26

**Authors:** Ikuo Watanobe, Yuzuru Ito, Eigo Akimoto, Yuuki Sekine, Yurie Haruyama, Kota Amemiya, Fumihiro Kawano, Shohei Fujita, Satoshi Omori, Shozo Miyano, Taijiro Kosaka, Michio Machida, Toshiaki Kitabatake, Kuniaki Kojima, Asumi Sakaguchi, Kanako Ogura, Toshiharu Matsumoto

**Affiliations:** ^1^Department of General Surgery, Juntendo University Nerima Hospital, 3-1-10 Takanodai, Nerima, Tokyo 177-8521, Japan; ^2^Department of Diagnostic Pathology, Juntendo University Nerima Hospital, 3-1-10 Takanodai, Nerima, Tokyo 177-8521, Japan

## Abstract

Duplication of the alimentary tract is a rare congenital malformation that occurs most often in the abdominal region, whereas esophageal duplication cyst develops typically in the thoracic region but occasionally in the neck and abdominal regions. Esophageal duplication cyst is usually diagnosed in early childhood because of symptoms related to bleeding, infection, and displacement of tissue surrounding the lesion. We recently encountered a rare adult case of esophageal duplication cyst in the abdominal esophagus. A 50-year-old man underwent gastroscopy, endoscopic ultrasonography, computed tomography, and magnetic resonance imaging to investigate epigastric pain and dysphagia that started 3 months earlier. Imaging findings suggested esophageal duplication cyst, and the patient underwent laparoscopic resection followed by intraoperative esophagoscopy to reconstruct the esophagus safely and effectively. Histopathological examination of the resected specimen revealed two layers of smooth muscle in the cystic wall, confirming the diagnosis of esophageal duplication cyst.

## 1. Introduction

As reported by Ladd and Gross, duplication of the alimentary tract is a rare congenital malformation that develops potentially anywhere in the gastrointestinal tract, from the root of the tongue to the anus [1]. Several theories have been suggested to explain the cause of duplication [2]. Popular theories include persistence of fetal gut diverticula, abnormal recanalization of the solid stage of development of the primitive gut, partial twinning, and a split notochord. We recently encountered a case of asymptomatic esophageal duplication cyst (EDC) that was not discovered until the age of 50 years. EDCs account for 20% of all the gastrointestinal duplication cysts [3]. In this case, the EDC in the lower esophageal region was treated laparoscopically because it was continuous with the mediastinum and abdominal cavity. The postoperative course was excellent. Here, we report this adult case of EDC and review the literature.

## 2. Case Presentation

A 50-year-old man had a history of an operation for lumbar herniated disc at the age of 37 and hypertension since the age of 42 that was controlled with medication. He visited a nearby clinic because of epigastric pain and dysphagia that started 3 months earlier. He was referred to our hospital because of gastroscopic findings of extrinsic compression.

**Figure 1:**
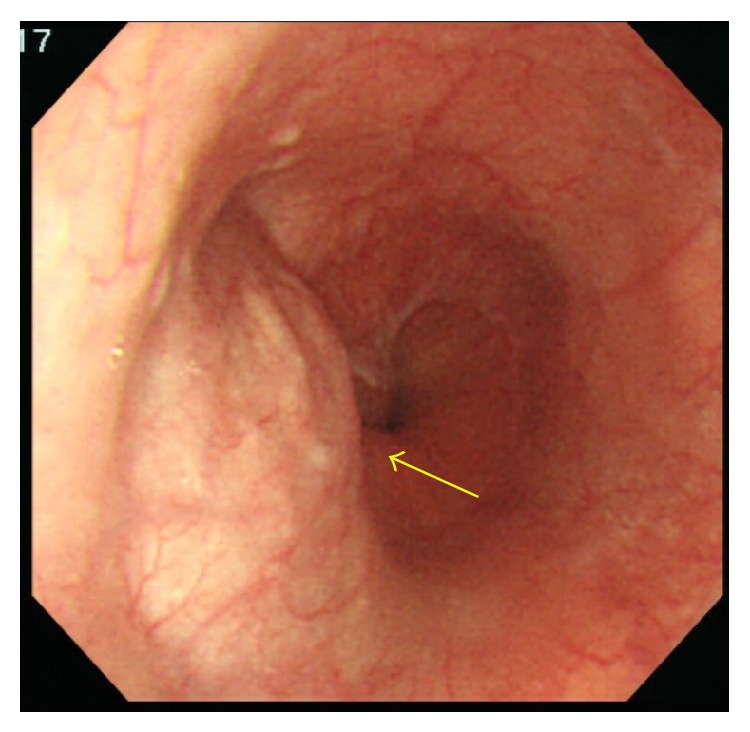
Transnasal gastroscopy. A submucosal tumor (arrow) of approximately 2 cm is visible at the 9 o'clock position in the lower esophagus, 41 cm from the tip of the nose. The surface of the tumor is smooth, and all the findings indicate gastrointestinal stromal tumor.

**Figure 2:**
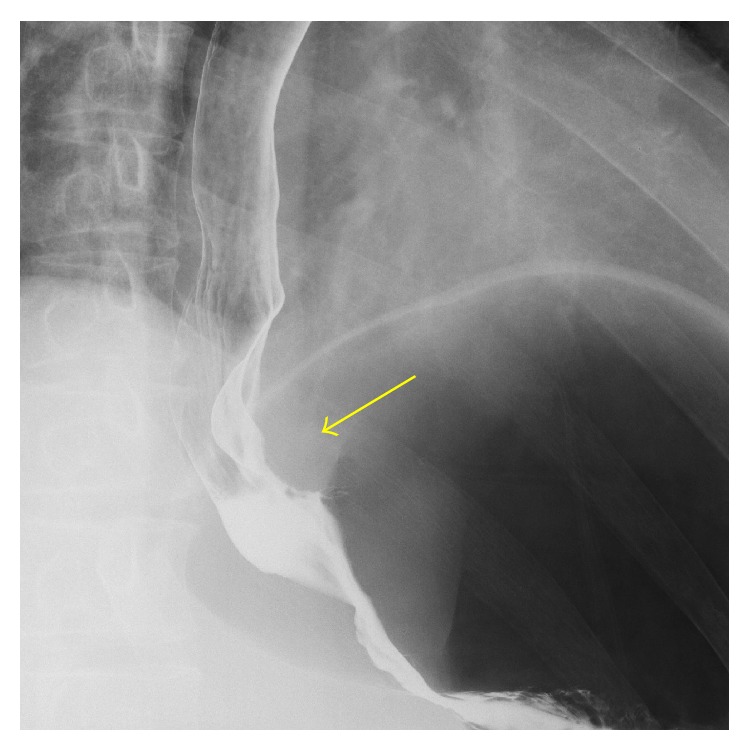
Barium esophagography. The image reveals extrinsic compression by a mass with a smooth surface (arrow) in the intra-abdominal esophageal region. Extension of the esophageal wall was good.

**Figure 3:**
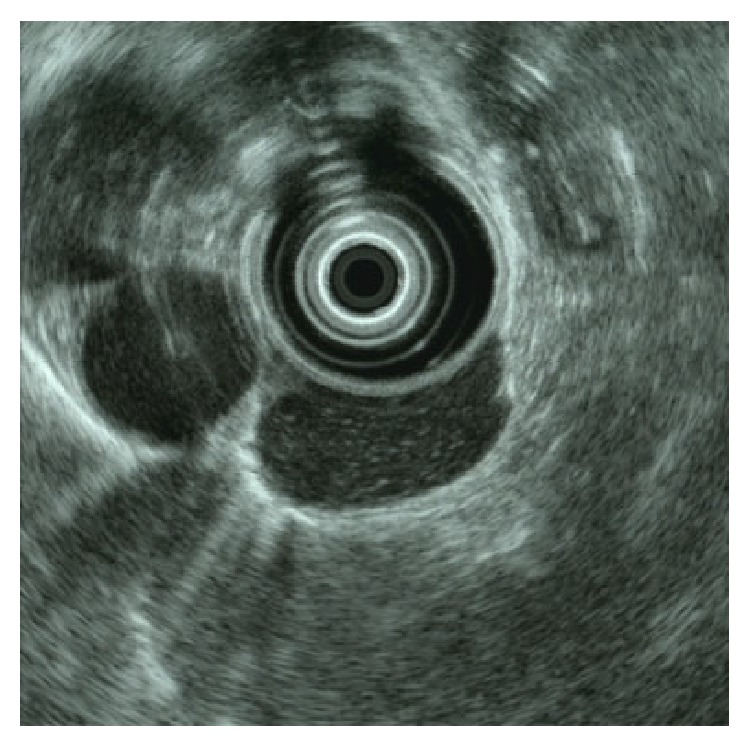
Endoscopic ultrasonography. The image shows a cystic mass extending from the lower esophagus to the cardiac region of the stomach and is filled with viscous components. No echoic debris indicative of bleeding or solid components was observed.

**Figure 4:**
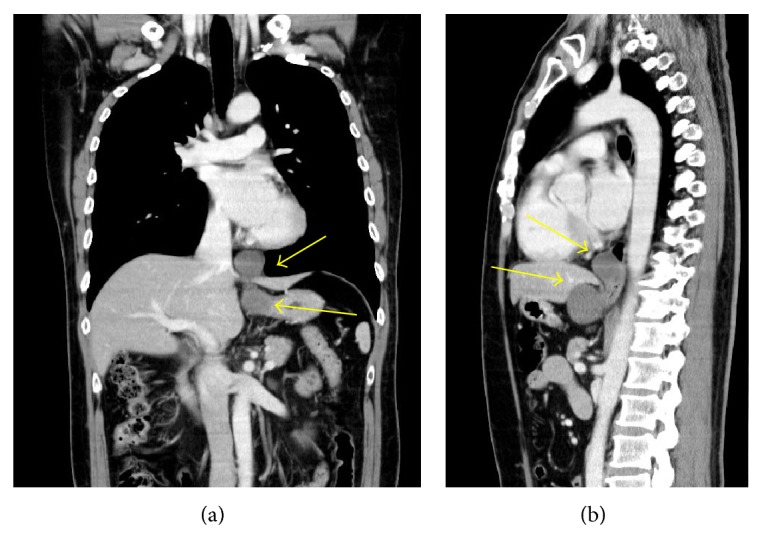
Computed tomography. The images reveal a dumbbell-shape iso-enhanced mass with a smooth surface, which extends parallel to the esophagus and spreads over and below the esophageal hiatus.

**Figure 5:**
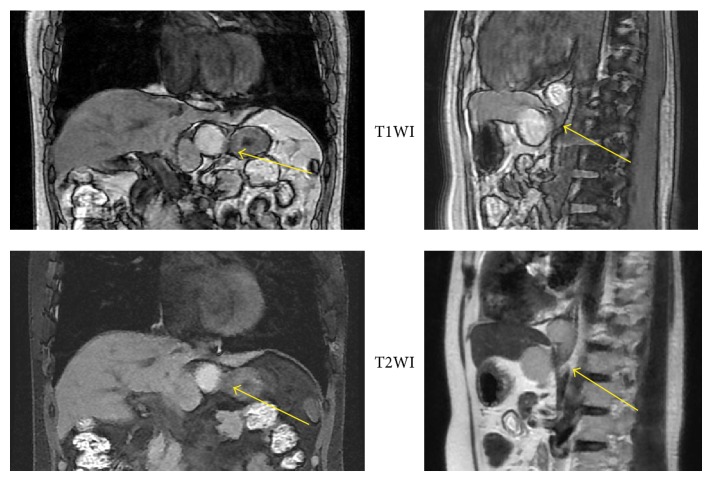
Magnetic resonance imaging. The images reveal a cystic mass extending from the lower esophagus to the cardiac region of the stomach. The mass appears to be located in or even outside the muscular layer and may contain mucin, high protein fluid, or blood.

The initial physical examination revealed normal heart and lung sounds, a flat and soft abdomen, and no tenderness on palpation. No superficial lymph nodes were palpable. Hematological and biochemical findings were normal. Gastroscopy performed at our hospital revealed a submucosal tumor with a smooth surface at the 9 o'clock position in the lower esophagus (Figure 1). Barium esophagogram showed extrinsic compression from the lower esophagus to the gastroesophageal junction (Figure 2). Good expansion and smooth mucosa were noted. Endoscopic ultrasonography showed a cystic mass in the esophageal wall extending from the lower esophagus to the cardiac region of the stomach (Figure 3), with the suspected presence of viscous fluid inside the cyst. Computed tomography (CT) showed an iso-enhanced dumbbell-shaped mass (3.5 × 3 cm) with a smooth surface and homogeneous content, which extended from the lower thoracic esophagus to the cardiac region of the stomach (Figure 4). Magnetic resonance imaging showed a mass that was hyperintense and moderately hyperintense on T1- and T2-weighted imaging, respectively, with and without fat suppression (Figure 5). Although gastrointestinal stromal tumor and leiomyoma were also suspected, the patient was diagnosed as having EDC based on imaging findings and underwent laparoscopic resection.

**Figure 6:**
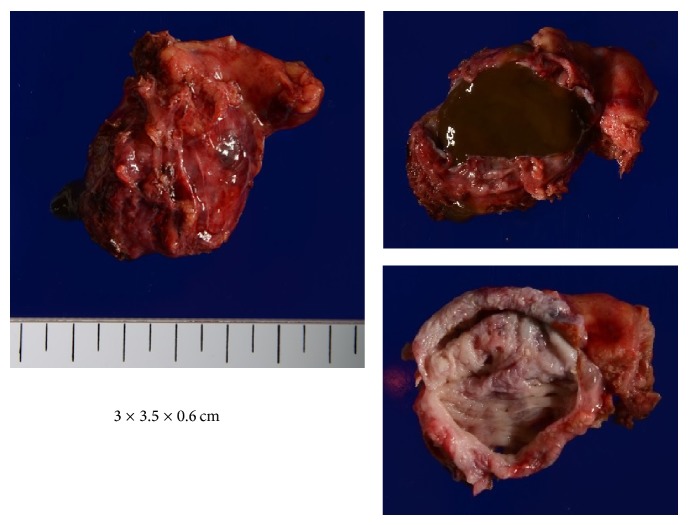
Resected specimen. The images show a 3 × 3.5 × 0.6 cm soft mass with no solid components. The content of the mass is a highly mucous, reddish brown fluid with no odor.

**Figure 7:**
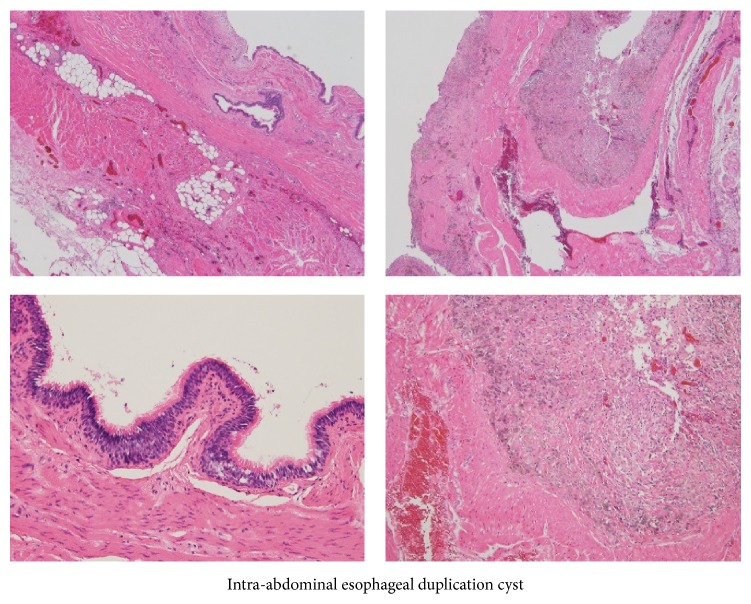
Histopathological examination. The wall of the mass is composed of two layers of smooth muscle fibers, and the cavity is lined with pseudostratified columnar epithelium. Bleeding and hemosiderin deposition are visible in certain areas of the cystic wall.

Intraoperatively, the mass was soft and elastic and had a smooth surface in the lesser curvature of the stomach near the cardiac region and along the esophagus when approached from the mediastinum by partially dissecting the crus of the diaphragm. The mass was carefully resected along the esophagus in the abdominal cavity toward the mediastinum. At the resection site, normal mucosa was left in some areas, but in other areas resection extended through all layers. Using a 3-0 synthetic absorbable suture, the surgical site was closed under intraoperative esophagoscopic observation to ensure proper closure and prevent esophageal stricture due to suturing. Cystic fluid in the resected specimen was mucous and reddish brown, with no cellular components (Figure 6). Histopathological findings revealed that the cyst consisted of two layers of smooth muscle and the inside of the cavity was lined with pseudostratified columnar epithelium (Figure 7). These findings, with no evidence of malignancy, led to the definitive diagnosis of EDC. The postoperative course was unremarkable, and the patient resumed a normal diet on postoperative day 4 and was discharged on postoperative day 10.

## 3. Discussion

Duplication of the alimentary tract is rare malformation observed in 1 of 25,000 deliveries [4]. In 1940, Ladd and Gross reported that diseases with common features but were named differently as enteric cyst, enterogenous cyst, giant diverticula, ileum duplex, and inclusion cysts were the same disease and should be collectively called duplication of the alimentary tract [1]. Duplications of the alimentary tract are hollow structures that have a muscular coat, usually composed of two layers, and are lined with epithelium similar to that found in the colon or some other portions of the gastrointestinal tract. These lesions are always contiguous to some portion of the alimentary tube, and they were strongly adherent to it in all but 1 reported case. The type of epithelium lining in the duplication cyst does not necessarily correspond to that in the alimentary tract to which it is attached. However, due to their morphological and histological variation, even duplications located away from the gastrointestinal tract and those with no muscular layers are now classified as alimentary tract duplication. The pathogenic mechanisms of EDC are unknown but are thought to be associated with abnormal esophageal development in the fifth to eighth weeks of gestation, when the posterior primitive foregut coalesces to form a single esophageal lumen. In more than 80% of cases, EDC is diagnosed before the age of 2 years when the patient experiences acute abdominal or bowel obstruction or other associated complications. A minority of cases remain asymptomatic until adulthood [5]. After bronchogenic cyst, EDC is the second most common benign posterior mediastinal lesion in children. EDC is relatively common in children presenting with mediastinal masses and accounts for 30% of all pediatric posterior mediastinal masses [6]. Differentiating between bronchogenic and esophageal cysts is more difficult because both derive from the foregut and contain ciliated epithelium. The difference between bronchogenic cysts and EDCs is that duplication cysts of bronchogenic origin do not have two layers of smooth muscle; instead they contain cartilage, bronchogenic glands, or both.

The present case is extremely rare because intra-abdominal EDC was discovered because of the onset of epigastric pain and dysphagia at the age of 50 years. A literature search extracted only 18 published case reports of intra-abdominal EDC [7–23], making the present case the 19th case (Table 1). In 7 of the previous 18 cases, EDC was incidentally diagnosed on CT scans that were taken as a part of comprehensive examination for other diseases. The most common complaint was pain (12 cases), followed by dysphagia. Interestingly, adults (*n* = 15) accounted for 79% of the patients with intra-abdominal EDC even though many duplication cysts of the alimentary tract are diagnosed before 2 years of age. This may be because no structures in the vicinity of the lower esophagus restrict the growth of tumor. EDC size ranged from 1 to 15 cm and no significant differences in patient background factors were observed between symptomatic patients and patients whose diagnosis was incidental. In general, the preoperative diagnosis of EDC is made based on CT and endoscopic ultrasonography findings. As in the present study, the definitive diagnosis of EDC is relatively easy for lesions with homogeneous signal intensity and smooth margins. However, it is sometimes difficult to definitively diagnose mediastinal cystic masses because of diverse components, such as hemorrhage, sebum or sebaceous fluid, and proteinaceous fluid. Surgical resection is recommended as the primary treatment for EDC because of reports of malignant transformation of cysts even though the frequency is unknown [24]. Although laparoscopic resection has been widely performed in recent years, it is essential to perform gross total resection because cysts can cause necrosis and fistula formation in nearby structures including the intestines and peritoneum [25] and because recurrence due to incomplete resection has been reported [26]. In our case, intraoperative esophagoscopy was performed after gross total resection of the EDC to ensure the accurate reconstruction of the esophageal wall, and this enabled us to verify the absence of postreconstruction esophageal stricture and to discover fragile areas in the esophagus due to surgical abrasion.

In summary, we reported an extremely rare adult case of intra-abdominal EDC and reviewed the 18 previously published case reports.

## Figures and Tables

**Table 1:** Published case reports of intra-abdominal esophageal duplication cyst.

	Reference	Year	Age	Sex	Symptoms	Location	Size (cm)	Treatment
(1)	Ruffin and Hansen [7]	1989	38	F	Epigastric pain, nausea, and vomit	Distal esophagus	4	Resection
(2)	Harvell et al. [8]	1996	57	F	Epigastric pain	Superior border of the body of pancreas	2.2	Laparoscopic resection
(3)	Karahasanoglu et al. [9]	1997	51	M	Dysphagia, weight loss, and epigastric pain	Subdiaphragm	11	Esophagogastrectomy
(4)	Janssen and Fiedler [10]	1998	56	F	Incidental (staging CT for rectal tumor)	Superior to the left kidney	8	Open resection
(5)	Rathaus and Feinberg [11]	2000	5	F	Epigastric pain	Between the left lobe of the liver and the cardia	1	Open resection
(6)	Nelms et al. [12]	2002	44	M	Low back pain	Diaphragmatic crura	7	Laparoscopic resection
(7)	Vijayaraghavan and Belagavi [13]	2002	70	F	Incidental (retching, giddiness, and headache)	Midline between the stomach and liver	7.5	Open resection
(8)	Noguchi et al. [14]	2003	26	F	Incidental (anal bleeding)	Right anterior wall of the distal esophagus	4	Laparoscopic resection with esophageal repair (Nissen)
(9)	Kin et al. [15]	2003	51	F	Incidental (staging CT for breast cancer)	Diaphragmatic crura	4.5	Laparoscopic resection with intraoperative esophagoscopy
(10)	Sakurai et al. [16]	2006	62	M	Dysphagia, upper abdominal pain	Bifurcation of the trachea through the proximal portion of the stomach	15	Resection, thoracotomy followed by laparotomy
(11)	Martin et al. [17]	2007	50	F	Left flank pain	Inferior portion of the pancreatic body/tail and the transverse mesocolon	6.5	Open resection
(12)	Martin et al. [17]	2007	60	M	Epigastric pain, gastric outlet obstruction	Dorsal to the second portion of the duodenum and the pancreatic head	10	Open resection
(13)	Aldrink and Kenney [18]	2011	2	M	Incidental (in laparoscopic fundoplication)	Anterior portion of the gastroesophageal junction	3	Laparoscopic resection with fundoplication
(14)	Gümüş et al. [19]	2011	18	F	Dyspeptic complaints	Lower end of the esophagus adjacent to the liver	4.2	Open resection
(15)	Bhamidipati et al. [20]	2013	69	M	Incidental (CT for diverticulitis)	Gastroesophageal junction	4.4	Laparoscopic resection
(16)	Pujar et al. [21]	2013	13	F	Pain in epigastric region	Gastroesophageal junction below the left lobe of the liver	5	Laparoscopic resection
(17)	Mori et al. [22]	2013	9	M	Incidental (CT for hematuria)	Ventral surface of the abdominal esophagus	2	Laparoscopic resection
(18)	Castelijns et al. [23]	2014	20	M	Nausea, colic pain	Gastroesophageal junction	3.2	Laparoscopic resection
(19)	Our case	**2014**	**50**	**M**	**Epigastric pain, dysphagia**	**Intra-abdominal esophagus extending to the distal thoracic esophagus**	**3.5**	**Laparoscopic resection with intraoperative esophagoscopy**

CT, computed tomography.
